# Dental Blogs, Podcasts, and Associated Social Media: Descriptive Mapping and Analysis

**DOI:** 10.2196/jmir.7868

**Published:** 2017-07-26

**Authors:** Julia Melkers, Diana Hicks, Simone Rosenblum, Kimberley R Isett, Jacqueline Elliott

**Affiliations:** ^1^ School of Public Policy Georgia Institute of Technology Atlanta, GA United States; ^2^ College of Computing Georgia Institute of Technology Atlanta, GA United States

**Keywords:** social media, blogs, podcasts, clinical information, dentistry

## Abstract

**Background:**

Studies of social media in both medicine and dentistry have largely focused on the value of social media for marketing to and communicating with patients and for clinical education. There is limited evidence of how dental clinicians contribute to and use social media to disseminate and access information relevant to clinical care.

**Objective:**

The purpose of this study was to inventory and assess the entry, growth, sources, and content of clinically relevant social media in dentistry.

**Methods:**

We developed an inventory of blogs, podcasts, videos, and associated social media disseminating clinical information to dentists. We assessed hosts’ media activity in terms of their combinations of modalities, entry and exit dates, frequency of posting, types of content posted, and size of audience.

**Results:**

Our study showed that clinically relevant information is posted by dentists and hygienists on social media. Clinically relevant information was provided in 89 blogs and podcasts, and topic analysis showed motives for blogging by host type: 55% (49 hosts) were practicing dentists or hygienists, followed by consultants (27 hosts, 30%), media including publishers and discussion board hosts (8 hosts, 9%), and professional organizations and corporations.

**Conclusions:**

We demonstrated the participation of and potential for practicing dentists and hygienists to use social media to share clinical and other information with practicing colleagues. There is a clear audience for these social media sites, suggesting a changing mode of information diffusion in dentistry. This study was a first effort to fill the gap in understanding the nature and potential role of social media in clinical dentistry.

## Introduction

Internet technology is changing the way clinical information is available to dental practitioners. Similar to the medical community, dental professionals rely, at least in part, on online resources to seek information relevant to their practice needs [1-6]. The notion of continual learning that facilitates knowledge currency is potentially enabled by broad and accelerated electronic access to information [7]. More recently, the adoption of interactive social media, such as blogs and discussion boards, has been shifting the way clinical professionals acquire and interact with relevant practice information [8].

Studies of social media in both medicine and dentistry have largely focused on the value of social media for marketing to and communicating with patients and for clinical education [1,3,5,6,8,9]. While these articles suggest that the use of social media is changing the environment of health communication [10,11], there is still much to be learned about the use of social media for communication between health care professionals [12]. Studies in medicine have begun to explore the application and importance of social media for increasing the rapidity and reach of clinical information to physicians, emergency medical personnel, and nurses [1,6,12-14]. We found only 1 such study in dentistry, of the Internet Dental Forum [15]. The aim of our study was to document the entry, growth, and content of blogs, podcasts, and associated social media sources disseminating clinical information to dentists and hygienists. We developed an inventory of blogs and podcasts (Multimedia Appendix 1) and categorized their content to assess the ways that the dental professional community initiates and uses social media to disseminate information for their peers and colleagues.

## Methods

Blogs and podcasts are forms of media, accessible to anyone, that provide a vehicle for extended discussion of a topic, such as would be required to convey useful information about advances in dental research to practicing dentists. Therefore, we constructed an inventory of blogs and podcasts aimed at dentists and hygienists. These sources all constitute public domain data available online, and therefore are exempt from institutional review board oversight. We did not include sources that may be clinically relevant but are membership platforms, such as Dentaltown, since they are not public domain data. Nor did we include blogs mounted on the Dentaltown platform.

Blogs and podcasts were inventoried through a combination of online Google Boolean searches (“dentist” OR “dental” OR dentistry” for dentist blogs and podcasts; “hygienist” OR “dental hygiene” for hygienist blogs and podcasts) and iterative searches of discovered sites, accompanying links, and existing curated lists of social media resources. This “snowball” search process [16] was conducted by a team of 3 researchers until the redundancy in sources led to saturation—that is, no new sources were being identified to add to the core population of social media aimed at dental practitioners. Each source was coded by 3 researchers for general content and language, with results checked for consistency. Inconsistent codes were rechecked by 2 additional team researchers to resolve any differences and were recoded accordingly. Our purpose in this exploratory work was to create general coding categories that would allow us to broadly distinguish content aimed at dental practitioners as a first step in assessing social media resources for dental clinicians. Therefore, content was coded as “patient oriented” if the communication was aimed to inform current and potential patients about the practice or clinical procedures; “clinically relevant” if it included any treatment or clinical information relevant to dental clinicians; or “management/profession” if it had no clinically relevant information and was limited to financial, marketing, or other aspects of the dental profession (Textbox 1). Using this inventory as a foundation, we searched for YouTube video channels, Facebook pages, and Twitter handles associated with the blog and podcast hosts. We excluded accounts if they were personal sites or appeared to communicate with patients only.

To verify that we had an unduplicated list of unique sources, we identified the host (individual’s name or names), their organizational affiliation, active practicing status, degree, and host type (practicing dentists and hygienists; consultants; companies, such as distributors or manufacturers; professional associations; or media). We eliminated duplicates to arrive at our final inventory of 89 hosts of 264 social media sources (51 blogs, 46 podcasts, 69 Twitter, 56 Facebook, and 42 YouTube). We then examined descriptive statistics on the range of modalities used (by host) and variation across the different groups of interest (by host type, audience, and content). Table 1 provides a description of the measures.

Of the social media resources in our inventory, blogs are most amenable to content coding due to their easily extractable text. Thus, as an additional step in our exploratory work, we extracted the raw text from the blog sites and used topic modeling to gauge and organize content. Our purpose was to explore the patterns and content of clinically relevant information disseminated to dental practitioners through social media. This technique identifies clusters of words that occur together, that is, in the same paragraph, relatively often. We gathered the raw text of 18,991 posts from the 24 blogs we had broadly categorized as clinically relevant into a database and performed text analysis using Wordstat 7 software (Provalis Research). First, we removed common words and reduced inflected or derived forms to a common root. Topics were identified in the text using a 2-stage factor analysis of a word-by-paragraph matrix in which words with a factor loading of 0.50 were retained. In the first stage, all blogs were analyzed, and the pattern of co-occurrence of topics in blogs was used to assign blogs to 1 of 2 groups based on topic similarity; 7 blogs did not cluster with others and were analyzed independently in the second stage. To identify the topics characterizing the posts of each group of blogs, a second factor analysis of the paragraph-by-word matrix was performed for each group separately. The results were lists of topics represented by words that frequently occurred together in paragraphs in blog posts.

**Content coding categories for content aimed at dental practitioners in blogs and podcasts.**

Clinically relevant

- Includes at least one post, episode, or video about clinically relevant topics, including patient care, dental procedures, dental materials, clinical technology, and use of dental or dental hygiene products.

Dental management/profession

- Discusses topics relevant to dental practice management or the dental or dental hygiene profession, practice management technology or software for office management exclusive of patient care, and does not include any reference to patient care or clinical issues.

**Table 1:** Dental social media modality measures.

Type	Measures	Description
**Blog**		
	Duration	Length of time between first post and most recent post
	Count	Number of blog entries
**Podcast**		
	Duration	Length of time between first episode and most recent episode
	Count	Number of podcast episodes
**Twitter**		
	Duration	Length of time between joining and most recent tweet
	Count	Number of tweets
	Followers	Number of Twitter followers
**Facebook**		
	Duration	Length of time between start of the Facebook page and most recent post
	Likes	Number of times the specific Facebook page has been “liked”
**YouTube**		
	Duration	Length of time between first uploaded video and most recent uploaded video on channel
	Uploads	Number of videos uploaded to the channel
	Subscribers	Number of individual subscriptions to the channel
	Views	Sum of the number of views of all uploaded videos on the channel

## Results

### Social Media Hosts and Modalities

Of the 264 social media accounts across all modalities aimed at the dissemination of clinical information to dentists, we found 89 unique social media hosts maintaining an average of 3 modalities (eg, a blog, Twitter account, and Facebook account). The largest group of hosts (49 hosts, 55%) were practicing dentists or hygienists, followed by consultants (27 hosts, 30%), media including publishers and discussion board hosts (8 hosts, 9%), and professional organizations and corporations, including Patterson Dental and DentalEZ (5 hosts, 6%).

**Figure 1:**
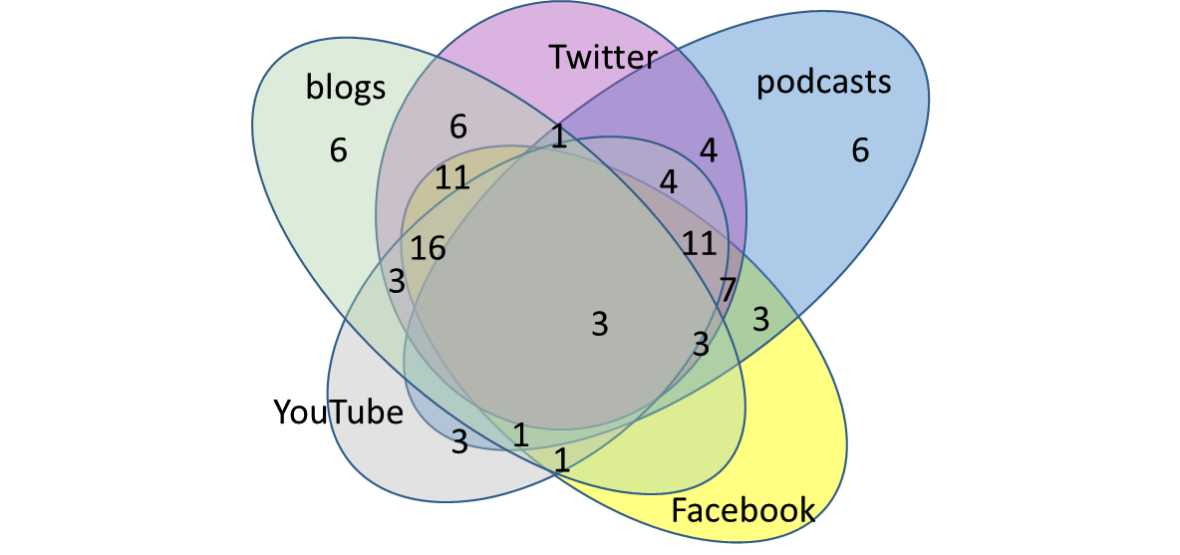
Social media modality combinations, by 89 hosts.

Blog and podcast hosts were relatively distinct, with only 8 hosts having both a blog and a podcast. Only 12 of the 89 hosts maintained just 1 platform, and 3 hosts used all 5 types of social media. The 5-way Venn diagram in Figure 1 shows how hosts used multiple modalities and how they overlapped.

**Table 2:** Number of social media sites targeted at dental clinicians, by host type and content.

Host type		Number	No. of which engaged in other social media			Share of accounts with clinically relevant content (all modalities) n (%)
			Facebook	Twitter	YouTube	
**Practicing dentists and hygienists**						
	Blog	20	9	13	6	38 (79)
	Podcast	24	12	17	8	28 (46)
	Both	1	1	1	0	0
**Consultants**						
	Blog	9	7	9	5	1 (3)
	Podcast	13	8	8	9	5 (13)
	Both	5	4	4	2	3 (15)
**Media**						
	Blog	5	5	5	2	16 (94)
	Podcast	0	0	0	0	0
	Both	1	1	1	1	4 (100)
**Professional associations**						
	Blog	3	3	3	3	7 (58)
	Podcast	0	0	0	0	0
	Both	1	1	1	1	3 (75)
**High-volume social media dentists**						
	Blog	4	2	4	3	7 (54)
	Podcast	0	0	0	0	0
	Both	0	0	0	0	0
**Companies**						
	Blog	2	2	2	2	8 (100)
	Podcast	1	1	1	1	1 (25)
	Both	0	0	0	0	0
**Totals by modality**						
	Blog	43	28	36	21	69 (57.5)
	Podcast	38	21	26	18	33 (33)
	Both	8	7	7	4	10 (32)
**Grand total**						
	Blog and podcast	97	56	69	43	112 (44.8)

Table 2 provides the number of hosts using each of the modalities. The table shows that practicing dentists and hygienists were much more likely than the others to just blog or podcast, but not engage in the other social media. In contrast, for consultants, media, associations, high-volume bloggers, and companies, blogging and podcasting seemed to be part of an overarching social media effort that more often than not included also Facebook, Twitter, or YouTube. The media and dental equipment companies were most engaged in providing clinical information. In contrast, consultants blogged and podcast about practice management for the most part. Practicing dentists tended to blog about clinical information, but their podcasts could be either clinically or management focused. Of the 4 high-volume dentists, 3 blogged and tweeted about clinically relevant information, but they used Facebook and YouTube for more management-oriented information.

### Social Media Duration and Presence in Dentistry

**Figure 2:**
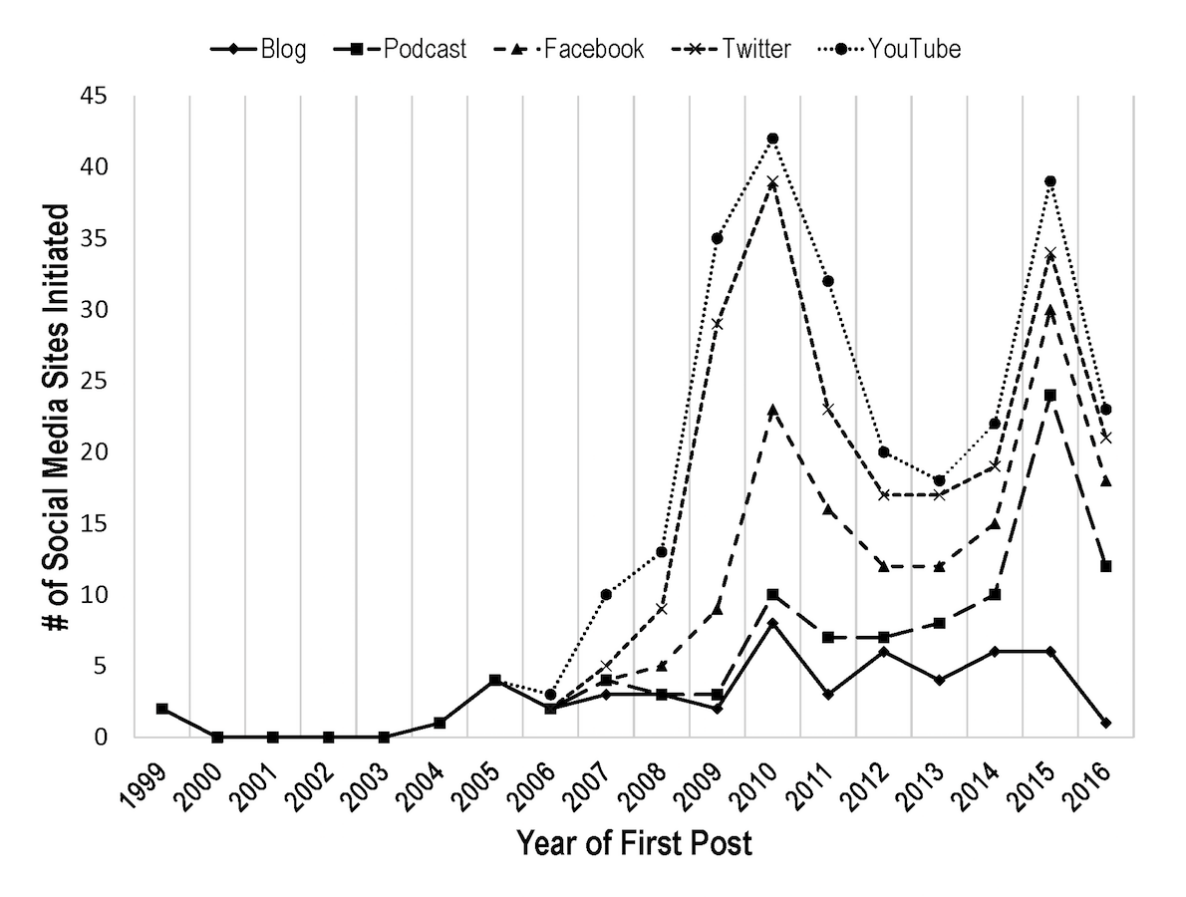
Year of initial social media post, by modality, for all hosts and modalities.

Social media emerged between 1999 and 2006: Facebook was launched in 2004, YouTube in 2005, and Twitter in 2006. Practicing dentists and consultants began to use these platforms within 2 years of their creation. While widespread podcast availability began in 2004 [17], the first dental podcast began in 2008. The first adopters of social media for communication with clinicians were practicing dentists, hygienists, and consultants, followed by industry and institutions, and, later, media organizations. While 2 consultants to the dental industry were active since 1999, most blogs in our dataset began around 2004-2005. Figure 2 provides a summary of the number of social media sites initiated by year in our dental inventory. Multimedia Appendix 2 provides a detailed timeline for all sites included in our analysis.

Hosting a social media site does not, however, indicate active use. We were limited to observing media that were active at or near the time we collected our data because discontinued blogs and other social media disappear (although some maintained their site without having posted for some time). Keeping in mind this caveat, Facebook was the least likely to disappear and YouTube the most. We found that 40% of YouTube sites (17/42 hosts), 71% of blogs (36/51 hosts), 72% of podcasts (33/46 hosts) and Twitter feeds (50/69 hosts), and 86% of Facebook pages (48/56 hosts) that we cataloged had posted within 2 months of our data collection in July 2016. About one-third of the discontinued offerings had been active for less than 11 months, and 16 of those for less than 1 month, suggesting experimentation with social media publishing. Both practicing dentists and consultants experimented. On the other hand, media outlets did not experiment but were also the latest to enter blogging or podcasting, starting 3 years later than practicing dentists on average. Every media blog and podcast we identified was still active at the time of this analysis.

Hosts also varied in how regularly they posted. Practicing dentists, not surprisingly, posted considerably less often than companies, media, and consultants. As Table 3 shows, practicing dentists averaged fewer than 10 blog posts per month, while the companies, media, and consultants posted well into double digits every month. This was not surprising, given the business development rationale for high visibility in the latter 3 groups. The 4 practicing dentists identified as high-volume posters stood out for posting up to 300 blog posts per month. They relied heavily on posting press releases or abstracts of journal articles to achieve this. These dentists are well-recognized speakers, so they may also have similar motivations to the other business-oriented groups. Notably, there was less variation in posting volume across the host types in low-effort Twitter posting. Conversely, practicing dentists were relatively active in posting videos and podcasts at about 5 per month, while high-volume social media dentists did not podcast. Howard Farran of Dentaltown was the most determined podcaster, averaging 26 posts per month. A total of 2 sites were initiated with a 1-time posting of a large amount of material on a single day, suggesting the use of social media as a type of repository.

**Table 3:** Social media presence: number and duration of postings.

Host type		Number	No. of months active	Posts per month, mean (SD)	Minimum	Maximum
**Blog**						
	Practicing dentist or hygienist	21	70	9.7 (19.9)	0.3	86.9
	Consultant	14	82	1.7 (1.4)	0.0	4.2
	Media	6	33	21.9 (31.4)	0.0	82.2
	Association	4	68	8.7 (5.8)	0.5	12.9
	High-volume social media dentist	4	101	92.4 (143.4)	16.3	307.5
	Company	2	35	58.6 (64.4)	13.0	104.1
**Twitter**						
	Practicing dentist or hygienist	31	52	26.3 (69.8)	0.3	371.4
	Consultant	21	69	71.7 (219.0)	0.0	1020.6
	Media	6	63	32.7 (16.6)	10.2	48.6
	Association	4	74	35.7 (17.0)	17.4	52.8
	High-volume social media dentist	4	102	47.3 (38.0)	3.5	95.1
	Company	3	87	45.9 (23.4)	27.4	72.3
**YouTube**						
	Practicing dentist or hygienist	14	41	4.5 (7.3)	0.0	27.4
	Consultant	16	54	1.7 (3.3)	0.1	13.5
	Media	2	24	7.6 (7.9)	2.0	13.2
	Association	4	104	1.5 (0.8)	0.9	2.6
	High-volume social media dentist	3	112	0.2 (0.2)	0.0	0.3
	Company	3	68	0.8 (0.4)	0.4	1.1
**Podcast**						
	Practicing dentist or hygienist	25	11	5.1 (7.7)	0.5	41.0
	Consultant	18	21	3.6 (N/A)	0.4	14.0
	Media	1	11	26.4 (N/A^a^)	26.4	26.4
	Association	1	55	0.3 (N/A)	0.3	0.3
	High-volume social media dentist	0	N/A	N/A (N/A)	N/A	N/A
	Company	1	15	1.2 (N/A)	1.2	1.2

^a^N/A: not applicable.

### Social Media Content

Hosts varied in the extent to which they provided clinical or practice-specific content. Looking across our 89 hosts, the 49 practicing dentists and hygienists and the 5 industry or association sites provided a balanced mix of clinical, professional, and management information across the platforms. In contrast, the 27 consultants were primarily focused on aspects of business practice, and the media outlets tended to prioritize clinical information. Only 2 of the 49 social media content sites published by practicing dentists or hygienists were exclusively clinical.

To probe the content of blogs in more detail, we topic modeled blogs containing at least some clinical content. Topics are lists of words that relatively frequently occurred in the same blog. In a topic model, each blog post can be a mix of topics, and each topic can be found in more than 1 blog post. The topics associated with each group of blogs were unique, but the sets of words overlapped. Following Ramage et al [18], we took advantage of this overlap to classify the topics into categories. Table 4 lists the categories, the number of topics associated with each category, and sample words drawn from topics in that category. Table 5 shows the number of topics in each category for each group of blogs or blog. Multimedia Appendix 3 displays the full list of topics

**Table 4:** Categories of topics with samples.

Categories		No. of associated topics	Sample words
**Leading topic**			
	Status/social	5	talk; thing; stuff; weekend
	Product announcement	2	visit; product; announce
**Dental care**			
	Clinical	37	odontogenic; nasal; sinusitis
	Materials or equipment	12	mill; impression; scanner
	Oral health	5	oral, health, bleach
**Dental practice related**			
	Company or organization	12	caesy; patterson
	Internet related	12	google; search; website; site
	Computers and imaging	9	drive; storage; backup; gb; hard
	Fees and payment	7	fee; provider; medicare; scholarship
	Management and employees	5	manager, team
	Conferences	3	booth; meeting; exhibit
**Other**			
	School	6	school, class
	Child	4	child; smile; kid; health
	Academic papers	4	study; publish; article; outcome
	Miscellaneous other	53	food, video, glide, marketing
Total		176	

In 4 of the cases, the strongest topic we labeled “status/social.” Many blogs shared a concern with the life side of work-life balance, and this is visible in the prominence of this topic. In the main group of 13 blogs, 73% (3414 posts) of posts contain at least one of the following words: guy, kid, talk, thing, stuff, weekend, kind, or couple. High-volume bloggers, in contrast, posted a lot of press releases, such as “announcements of products from industry leaders”—all words that appeared in their leading topic. Interestingly, the high-volume bloggers’ version of the status/social topic (their second strongest), instead of words referring to other people, contained “I’m” and “I’ve,” suggesting a focus on self as the authors perhaps sought to build themselves into brands. The DentalEZ blog’s lead topic differed, suggesting many announcements inviting readers to “stop” in at an upcoming “meeting” where they will have a “booth” on the “exhibit floor.”

While nonclinical content was a unifying factor across the blogs, clinical topics distinguished blogs from each other. The clinical words were so specialized and varied that blogs with a clinical focus did not cluster together. For example, Jablow, a high-volume poster with a lot of clinical content, separated from the other high-volume bloggers. Jablow, Lee Ann Brady, NYC Dentist, and Endo Blog each had at least 8 clinically relevant topics, and they remained singletons, not clustering with each other.

Issues associated with managing a practice were broadly discussed by many blogs. These issues include money (ie, fees, payment, and scholarships for students); teams and employees; websites and social media; brand names; conferences; and computer equipment (a favorite of high-volume bloggers). The topics were rounded out with a sprinkling of discussion of dental school, of children, of academic papers in blogs that posted abstracts of journal articles such as Jablow, and miscellaneous weak single-word topics.

**Table 5:** Number of topics in each category by blog.

Categories		Main group	High volume	Jablow	DentalEZ	New Dentist Now	Lee Ann Brady	NYC Dentist	Endo blog	Voice of Dental Ed	Total
Blogs		13	4	1	1	1	1	1	1	1	24
Posts		4677	9122	3007	658	522	478	318	193	16	18,991
**Leading topic**											
	Status/social	1	1	0	0	1	1	0	0	1	5
	Product announcement	0	1	1	0	0	0	0	0	0	2
**Dental care**											
	Clinical	1	1	7	1	0	11	6	10	0	37
	Materials or equipment	1	2	5	2	0	0	0	2	0	12
	Oral health	1	0	1	0	0	1	2	0	0	5
**Dental practice related**											
	Company or organization	1	2	5	3	1	0	0	0	0	12
	Internet related	3	3	1	1	2	1	1	0	0	12
	Computers and imaging	0	8	0	0	0	0	1	0	0	9
	Fees and payment	1	0	0	0	3	0	1	2	0	7
	Management and employees	2	1	0	1	1	0	0	0	0	5
	Conferences	0	0	0	2	1	0	0	0	0	3
**Other**											
	School	1	0	1	1	0	0	1	0	2	6
	Child	1	0	1	1	1	0	0	0	0	4
	Academic papers	0	0	2	1	0	0	0	1	0	4
	Miscellaneous other	6	4		8	1	4	9	8	13	53
Total		13	19	24	13	10	14	12	15	3	176

### Social Media Audience and Engagement

Ultimately, the question of whether practicing dentists and hygienists access these social media sites was central to understanding their impact. Our ability to ascertain the audience, or potential dental practitioner users, of the clinically relevant information provided through social media was, however, limited. Detailed access (“visits”), click, and download data for blogs and podcasts are typically available only to the host. However, using publicly available statistics, we were able to roughly estimate the potential reach of Facebook, Twitter, and YouTube. From these statistics, we found that the number of Facebook page likes was the largest, followed by Twitter followers, and then YouTube subscribers. Comparing across host types, practicing dentists and hygienists collectively had an audience size for YouTube that was about average for typical users of that modality, but a below-average audience size for Twitter and Facebook (Table 6). The average audience on Twitter was larger for dental management/profession topics, while the audience on Facebook was larger for clinically relevant topics.

As a caveat, these numbers demonstrate observable audience, but nothing more. Likes and followers can be bought, and we assumed that institutions with concerns for their business model and brand image might be more likely to invest in robot followers or likes. Comparing audiences directly across social media modalities was not reasonable, as subscription and followership behavior is different across these platforms. Further, liking and subscribing is a 1-click action and we could not assume continued active engagement going forward. Nor did we know who that audience was, which posts were viewed, or which posts were shared or forwarded.

**Table 6:** Average audience size by host type and content.

Host type	Type of content	Facebook page likes	Twitter followers	YouTube subscribers
**Company**				
	Clinically relevant	3703	11,207	149
	Dental management/profession	0	2346	34
**Consultant**				
	Clinically relevant	640	30	47
	Dental management/profession	1768	2711	138
**High-volume social media dentist**				
	Clinically relevant	0	2923	11
	Dental management/profession	4995	19,491	8
**Media**				
	Clinically relevant	25,685	7103	2808
	Dental management/profession	0	0	9
**Practicing dentist or hygienist**				
	Clinically relevant	15,528	398	775
	Dental management/profession	684	3292	102
**Professional association**				
	Clinically relevant	84,541	3195	2178
	Dental management/profession	0	21,703	1012

## Discussion

Our study showed that clinically relevant information is posted by dentists and hygienists on social media. To our knowledge, this study provided the first inventory and descriptive analysis of dental social media hosts and platforms. In the dental profession, dentist-to-dentist information distribution has always taken place formally at professional meetings and in study groups, and informally among colleagues. However, social media provide an opportunity for willing clinicians to share their tacit and earned wisdom across geography and time, suggesting a fundamental shift in the way information can be acquired by dental professionals. While clinical evidence will no doubt continue to be sourced from peer-reviewed research, how clinicians access and interact with that information—from others via online trusted sources rather than the pages of a journal—may be changing. Social media researchers note that “traditional media drove reach, while social media created intimacy and engagement” [19]. The familiarity or intimacy of social media hosts helps to build learning communities within the profession, extending collegial support systems (such as study groups) to the online format. Our topic analysis suggested that part of the power of the medium, in comparison with more formal channels, is the discussion of the full context of professional work (personal and professional). This is consistent with Burnett’s “information neighborhoods,” where professionals will wander around familiar and comfortable places (blogs, Twitter feeds, etc) and “bump into” useful information for which they were not explicitly looking or did not know they needed [20]. For dental practitioners, we showed that these neighborhoods include clinical information, but also a range of other resources relevant to practice management and the profession. So, a search for a treatment approach or solution may also lead to the discovery of something related to practice management or professional development.

With the growth of online access and resources, researchers have studied how, where, and why clinicians access both traditional and electronic resources [1,5,9,20-24]. These studies have laid the groundwork for understanding the relative attraction and use of online sources. The fact that most of the social media platforms are less than a decade old, and that the dental community has been active on these platforms for even fewer years, suggests that use of these resources will continue to evolve. Thus, published studies in recent years may not have captured the shift in interest in online resources, particularly among younger dentists and hygienists [2,4,21,23-32].

As social media continue to evolve, and a younger generation of dental clinicians move into practice, use of social media can be expected to increase. Spallek and colleagues [33] argued that the issue is not whether clinicians will use social media for professional use (because this is certain), but rather *how* social media information transmission benefits can be maximized. Perhaps the most striking finding of our research is the phenomenon of clinicians as information providers for other clinicians on social media. Clinician-to-clinician information dissemination likely fills a gap that many clinicians feel between dental research and the day-to-day context of practice [34]. Like Landry [35], we do not expect these social media resources to substitute for other traditional sources, but rather to supplement them. The professional trust and familiarity that develops in social media-enabled networks can create an identifiable source for rapidly disseminated, reliable information among professionals, which may be creating an important change in the information landscape [36,37].

Our study has limitations, but also presents opportunities for future work. In terms of limitations, social media are dynamic and there may be relevant blogs or podcasts that we have inadvertently excluded. The findings of this study present several questions relevant for future inquiry. Methodologically, our exploratory categorization and topic modeling illustrates the complexity of studying clinically specific information diffusion. Terms vary, and in these modalities are intertwined with other nonclinical content. Future research may include the development of topic dictionaries that allow for finer coding and categorization of clinical content in dentistry. For example, natural language processing has been helpful in other studies and may have application here as well [38]. In terms of scope, our study did not address how dental clinicians locate, select, or make use of information that is available on these blogs, podcasts, and other social media. Nor do we know how the social media hosts selected the information to be provided, the quality of the information provided, or its relevance to clinical practice. We do not understand the cumulative effects on the perceived value or quality of the information based on the overall visibility and involvement of dental social media hosts in the dental community. However, our work has developed a foundation to address the important implementation and clinical translation of science to practice issues relevant to social media in dentistry.

Our study demonstrated the participation of practicing dentists and hygienists in the social media environment as a way to share clinical and other information with practicing colleagues. We identified the use of different multimedia and text-based social media platforms for reaching dental practitioners. While this study was exploratory, we hope that it may provide an early indication of the potential that social media may have to develop and strengthen learning communities in dentistry and advance the uptake of current clinical information by practitioners. For dentists, it provides insight into the availability and providers of clinically relevant information in social media.

## Appendix

Multimedia Appendix 1 Blog and podcast inventory.

Multimedia Appendix 2 Timeline of dental social media.

Multimedia Appendix 3 Topic model of dental blogs.
