# Supplementary material for: Dental Blogs, Podcasts, and Associated Social Media: Descriptive Mapping and Analysis
Source: J Med Internet Res. 2017 Jul 26;19(7):e269. doi: 10.2196/jmir.7868 (PMC5553003; doi:10.2196/jmir.7868)
Supplement: Multimedia Appendix 1 [file jmir_v19i7e269_app1.pdf]

BLOG TITLE	BLOG URL	PODCAST TITLE	PODCAST URL
**AIR FORCE DENTISTRY FROM COLLEGE TO CAPTAIN**	http://usafdds.blogspot.com		
**ASK DR.SPINDEL**	http://lspindelnycdds.blogspot.com/		
**D IS FOR DENTIST**	http://www.disfordentist.com/		
**DENTAL BUZZ**	http://www.dentalbuzz.com/bloglist/		
**DENTAL MARKETING**	http://www.davidschwab.com/blog/	**THE PERSONAL REPORT: DENTAL PRACTICE PODCAST**	https://itunes.apple.com/us/podcast/personal-report-dental-practice/id1084236369?mt=2
**DENTAL REALIST**	https://dentalrealist.com/blog/	**DENTAL REALIST PODCAST**	https://itunes.apple.com/us/podcast/dental-realist-podcast/id1080244642?mt=2
**DENTALBLOGS.COM**	http://dentalblogs.com/		
**DENTALEZ**	http://www.dentalez.com/blog		
**DENTALTOWN**	http://www.dentaltown.com/Dentaltown/blogs.aspx	**DENTISTRY UNCENSORED HOWARD FARRAN**	https://itunes.apple.com/us/podcast/dentistry-uncensored-howard/id916907356?mt=2
**DENTECH BLOG: MARTY JABLOW DMD DENTAL NEWS AND TECHNOLOGY BLOG**	http://dentechblog.blogspot.com		
**DENTISTRYIQ (PENNWELL CORPORATION)**	http://www.dentistryiq.com/blogs.html		
**DR. PAUL'S DENTAL TECHNOLOGY**	http://computersindentistry.blogspot.com/		
**EMMOTT ON DENTISTRY**	http://emmottontechnology.com/		
**EXCEPTIONAL DENTAL PRACTICE MANAGEMENT**	http://dentalpracticemanagement.typepad.com/		
**HYGIENETOWN**	http://www.hygienetown.com/Hygienetown/blogs.aspx		
**INSIDE DENTAL TECHNOLOGY**	http://www.dentalaegis.com/idt/		
**INSIDE DENTISTRY**	https://www.dentalaegis.com/id/blog		
**INSPIREDHYGIENE**	http://inspiredhygiene.com/blog		
**JESSICA, CANDI AND MELIA**	http://www.hygieneedge.com/blog/		
**JOHN FLUCKE'S DENTAL TECHNOLOGY BLOG**	http://blog.denticle.com		
**LANMARK360**	http://www.lanmark360.com/blog		
**LEEANN BRADY**	leeannbrady.com		
**MARK FRIAS, RDH**	http://markrdh.com/		
**MOUTHING OFF**	http://www.asdablog.com/		

BLOG TITLE	BLOG URL	PODCAST TITLE	PODCAST URL
**MYFLOSSOPHY**	https://myflossophy.com/		
**NEW DENTIST NOW**	http://newdentistblog.ada.org/	**EVIDENCE-BASED DENTISTRY CHAMPION**	https://itunes.apple.com/us/podcast/evidence-based-dentistry-champion/id904406954?mt=2
**NEXT STEP DENTAL RESOURCE**	http://nextstepdentalresource.com/category/missys-blog/		
**NYC DENTISTS**	http://www.nycdentist.com/blog/		
**OFF THE CUSP**	http://www.offthecusp.com/		
**ONE LOOSE TOOTH**	http://oneloosetooth.com/blog/		
**ORTHOTOWN**	http://www.orthotown.com/Orthotown/blogs.aspx		
**PAUL HUNG DDS MS LIFE AS A DENTIST IN DALLAS FORTH WORTH**	http://nyucddds2013.blogspot.com/		
**PEDIATRIC DENTISTRY/ CYBERDENTIST**	http://cyberdentist.blogspot.com/		
**SALLY MCKENZIE**	http://www.mckenziemgmt.com/mBlog/	**THE NEW DENTIST**	https://itunes.apple.com/us/podcast/the-new-dentist/id342534574?mt=2
**T2 CONSULTING**	http://www.tedtakahashi.com/blog-3/		
**TAKACS LEARNING CENTER**	http://www.takacslearningcenter.com/category/thriving-dentist-show/	**THRIVING DENTIST SHOW WITH GARY TAKACS**	http://www.stitcher.com/podcast/thriving-dentist-show-with-gary-takacs-by-gary-takacs
**THE BLOGGING DENTIST**	http://thebloggingdentist.com/		
**THE CURIOUS DENTIST**	http://thecuriousdentist.com/		
**THE DAILY GRIND**	http://agdblogs.blogspot.com/		
**THE DENTAL GEEK**	http://www.thedentalgeek.com		
**THE DENTAL WARRIOR**	http://thedentalwarrior.com/		
**THE DENTISTMONEY BLOG**	http://dentistadvisors.com/blog/	**DENTIST MONEY**	https://itunes.apple.com/us/podcast/dentist-money/id1063782520?mt=2
**THE DIGITIAL DENTIST**	http://thedigitaldentist.com/		
**THE ENDO BLOG**	http://www.theendoblog.com/		
**THE MADOW BROTHERS**	http://www.madow.com/blog/		
**THE TAO OF DENTISTRY**	http://taoofdentistry.com/blog/		
**THE TOOTH BOOTH DENTAL BLOG/DENTALDUDE**	http://dentaldude.blogspot.com		
**THE VOICE OF DENTAL EDUCATION**	http://www.adea.org/GoDental/Dental_Blogs/Hear_from_Practitioners.aspx		
**THE WEALTHY DENTIST**	http://www.thewealthydentist.com/blog/		
**UNLOCK THE PPO**	http://www.unlocktheppo.com/blog/		

BLOG TITLE	BLOG URL	PODCAST TITLE	PODCAST URL
		**DENTURE SUCCESS | DENTURES | BEST DENTURE PRACTICES**	https://itunes.apple.com/us/podcast/denture-success-dentures-best/id1079897195?mt=2
		**A TALE OF TWO HYGIENISTS WITH MICHELLE AND ANDREW**	http://www.stitcher.com/podcast/off-the-cusp-podcast
		**ACCIDENTAL GENIUSES**	http://www.stitcher.com/podcast/accidental-geniuses
		**BLATCHFORD SOLUTIONS PODCAST | BUSINESS COACHING FOR DENTISTS**	http://www.stitcher.com/podcast/blatchford-solutions-podcast
		**BUSINESS OF DENTISTRY**	https://itunes.apple.com/us/podcast/business-dentistry-dr.-russell/id1038531011?mt=2
		**DENTAL DOC TALK**	https://itunes.apple.com/us/podcast/dental-doc-talk/id430391152?mt=2
		**DENTAL IMPLANT COWBOY**	https://itunes.apple.com/us/podcast/dental-implant-cowboy/id446994226?mt=2
		**DENTAL MARKETING MASTERY**	http://www.stitcher.com/podcast/dental-marketing-mastery
		**DENTAL MARKETING RADIO**	https://itunes.apple.com/us/podcast/localsearchfordentists.com/id614770624?mt=2
		**DENTAL PRACTICE 911 RADIO (FORMERLY THE WHOLE TOOTH)**	https://itunes.apple.com/us/podcast/dental-practice-911-radio/id386883768?mt=2
		**DENTAL PRACTICE MANAGEMENT | BETTER DENTISTS, HYGIENISTS, PATIENTS &...**	https://itunes.apple.com/us/podcast/dental-practice-management/id584153001?mt=2
		**DENTAL SOFTWARE ADVISOR**	https://itunes.apple.com/us/podcast/dental-software-advisor/id959770559?mt=2
		**DENTAL TALK RADIO FOCUSES ON THE CLINICAL, PRACTICAL AND BUSINESS OF DENTISTRY WITH CLINICIANS AND INDUSTRY EXPERTS AS GUESTS.**	https://itunes.apple.com/us/podcast/dental-talk-radio/id364086648?mt=2
		**DENTAL UP PODCAST**	https://itunes.apple.com/us/podcast/dental-up/id999909601?mt=2

BLOG TITLE	BLOG URL	PODCAST TITLE	PODCAST URL
		**DENTIST BRAIN CANDY**	http://www.stitcher.com/podcast/bryan-2/dentist-brain-candy
		**DENTISTRY'S IDEAL PRACTICES WITH JAYME AMOS**	https://itunes.apple.com/us/podcast/dentistrys-ideal-practices/id997844691?mt=2
		**DENTISTS, IMPLANTS AND WORMS**	http://www.stitcher.com/podcast/dentists-implants-and-worms
		**DR. CHRIS GRIFFIN SHOW: SIMPLE PRACTICE BREAKTHROUGHS TO MAKE YOUR LIFE...**	http://www.stitcher.com/podcast/dr-chris-griffin-show
		**FAST THOUGHTS ON DENTAL MARKETING**	https://itunes.apple.com/us/podcast/fast-thoughts-on-dental-marketing/id530145811?mt=2
		**HDIQ PODCAST**	https://itunes.apple.com/us/podcast/the-hdiq-podcast/id311656236?mt=2
		**PRACTICING WITH THE MASTERS**	http://www.stitcher.com/podcast/practicing-with-the-masters
		**SMILE SOURCE**	https://itunes.apple.com/us/podcast/smile-source-radio/id990044813?mt=2
		**T BONE SPEAKS PODCAST**	https://itunes.apple.com/us/podcast/t-bone-speaks-podcast/id1084246936?mt=2
		**THE 8E8 DENTAL MARKETING SHOW**	http://www.stitcher.com/podcast/the-8e8-dental-marketing-show
		**THE DELIVERING WOW DENTAL PODCAST**	http://www.stitcher.com/podcast/delivering-wow
		**THE DENTAL GUYS**	https://itunes.apple.com/us/podcast/the-dental-guys/id1052597962?mt=2
		**THE DENTAL HACKS PODCAST**	http://www.stitcher.com/podcast/the-dentalhacks-podcast
		**THE DENTAL INSIDERS**	https://itunes.apple.com/us/podcast/the-dental-insiders-podcast/id1059229774?mt=2
		**THE DENTALPRENEUR PODCAST W/ DR. MARK COSTES – BECOME MORE PROFITABLE...**	http://www.stitcher.com/podcast/the-dentalpreneur-podcast
		**THE DENTIST FREEDOM BLUEPRINT**	https://itunes.apple.com/us/podcast/dentist-freedom-blueprint/id974276340?mt=2

BLOG TITLE	BLOG URL	PODCAST TITLE	PODCAST URL
		**THE EXPLORER WITH DR. ALYSSA MARSHALL**	http://www.stitcher.com/podcast/the-explorer-with-dr-alyssa-marshall
		**THE MOLAR MAFIA DENTAL PODCAST WITH JERRY NEWMAN**	http://www.stitcher.com/podcast/the-molar-mafia-dental-podcast
		**THE PASSIONATE DENTIST PODCAST WITH DR. B. SAIB**	http://www.stitcher.com/podcast/the-passionate-dentist
		**THE RELENTLESS DENTIST SHOW WITH DRS. DAVID & KARAH MALOLEY**	http://www.stitcher.com/podcast/the-relentless-dentist
		**THE SNARKY DENTISTS**	http://www.stitcher.com/podcast/the-snarky-dentist
		**TOTALLY ORAL PODCAST**	http://www.stitcher.com/podcast/totally-oral
		**YOUR 30 DAY DENTAL MBA BY HOWARD FARRAN**	https://itunes.apple.com/us/podcast/howard-farran-dds-mba/id567540330?mt=2
		**YOUR DENTAL MARKETING DEPARTMENT**	https://itunes.apple.com/us/podcast/your-dental-marketing-department/id619472494?mt=2
		**YOUR DENTAL SUCCESS PODCAST: DIGITAL MARKETING FOR DENTISTS | SEO | DENTAL...**	http://www.stitcher.com/podcast/your-dental-success
