# Supplementary material for: Dental Blogs, Podcasts, and Associated Social Media: Descriptive Mapping and Analysis
Source: J Med Internet Res. 2017 Jul 26;19(7):e269. doi: 10.2196/jmir.7868 (PMC5553003; doi:10.2196/jmir.7868)
Supplement: Multimedia Appendix 2 [file jmir_v19i7e269_app2.pdf]

DETAILED TIMELINES

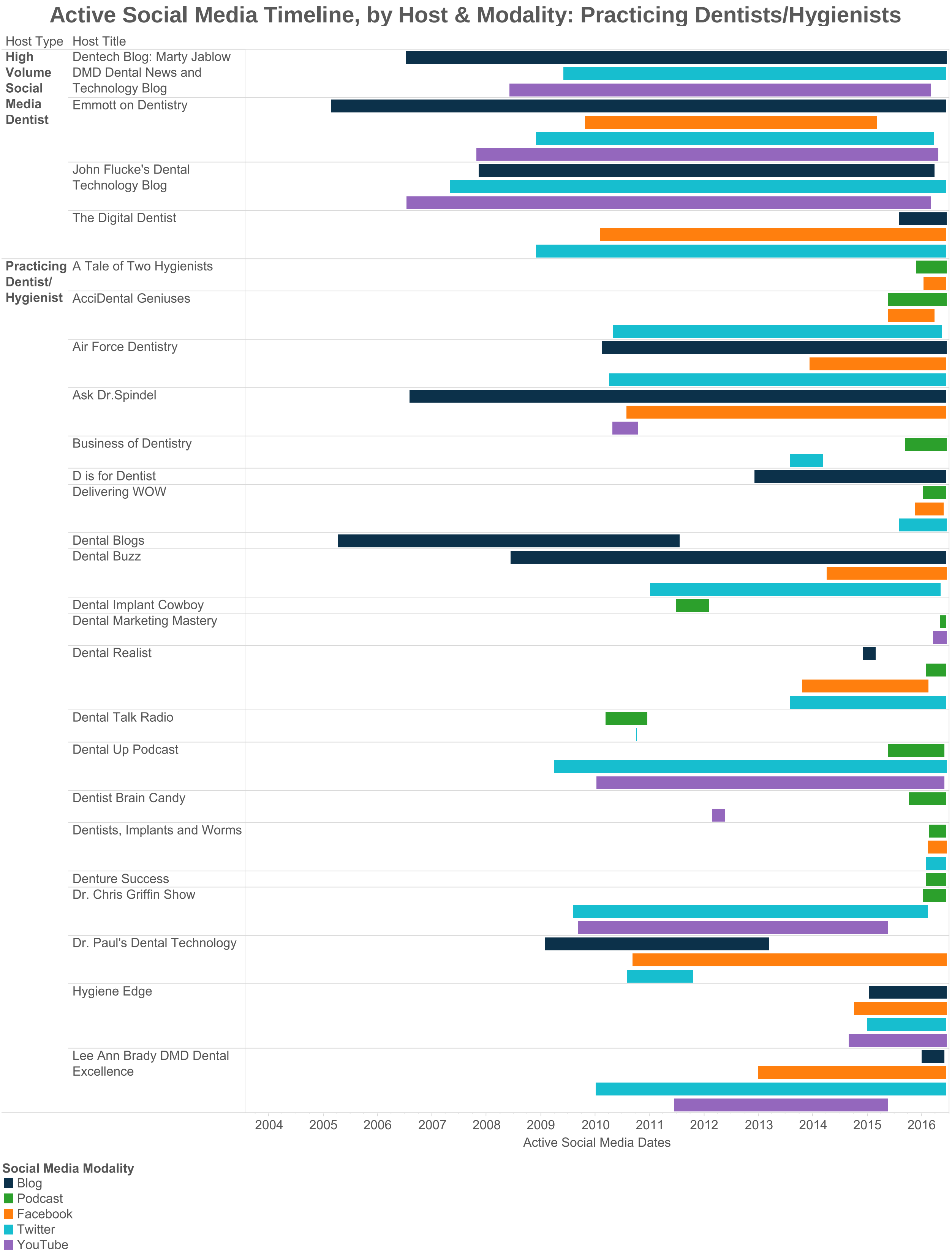

Active Social Media Timeline, by Host & Modality: Practicing Dentists/Hygienists Cont'd

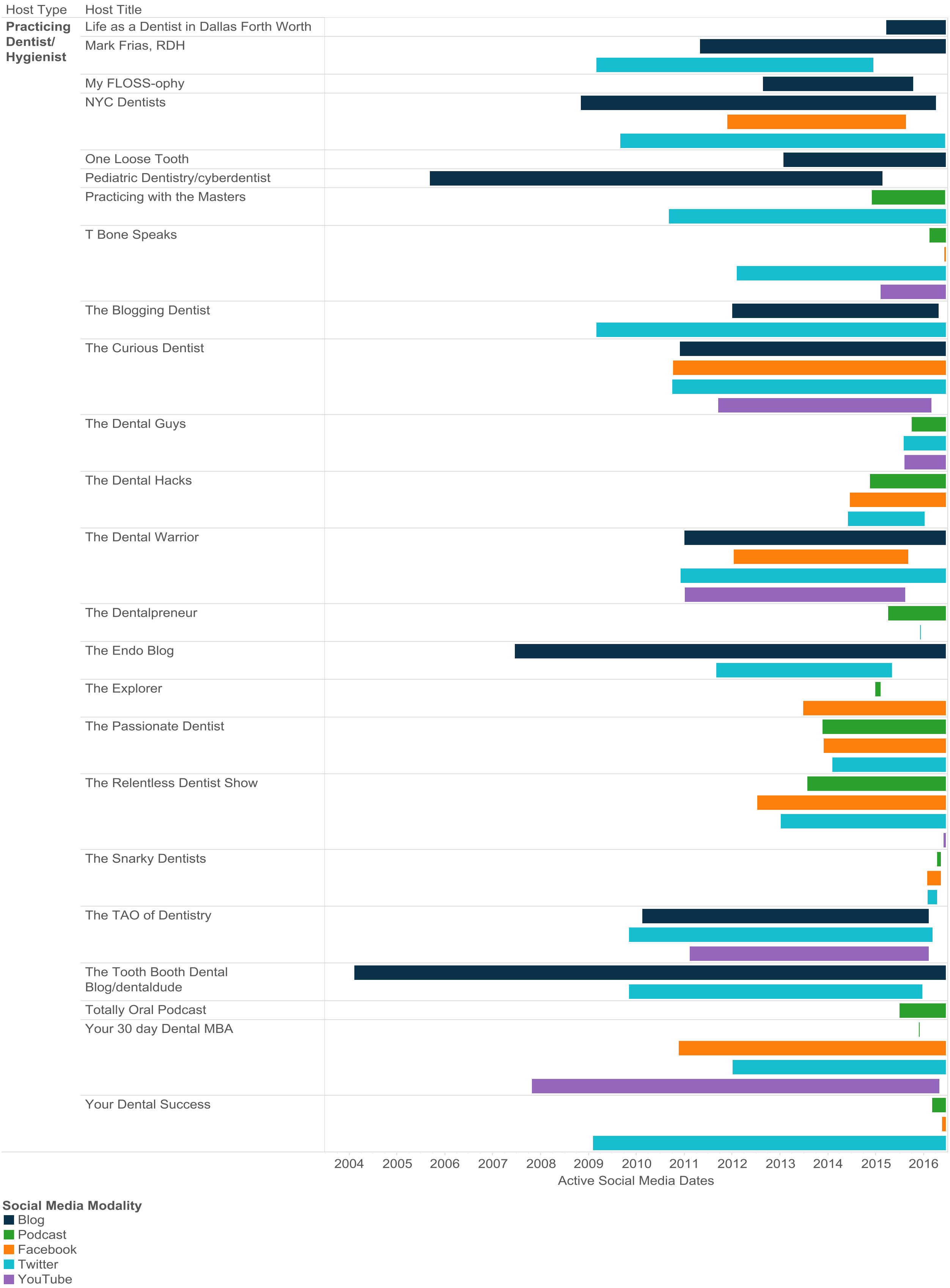

Active Social Media Timeline, by Host & Modality: Companies, Media, & Professional Assoc.

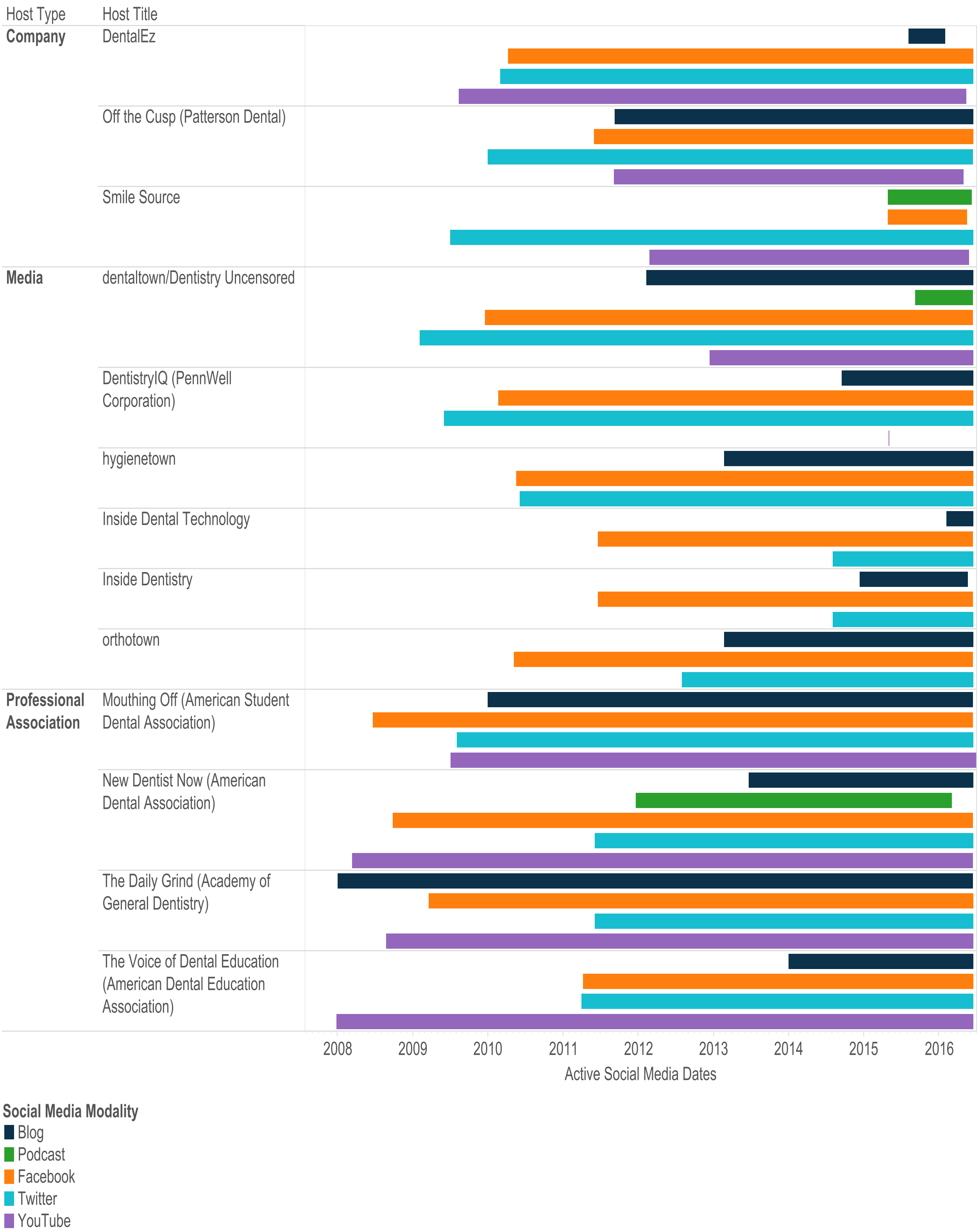

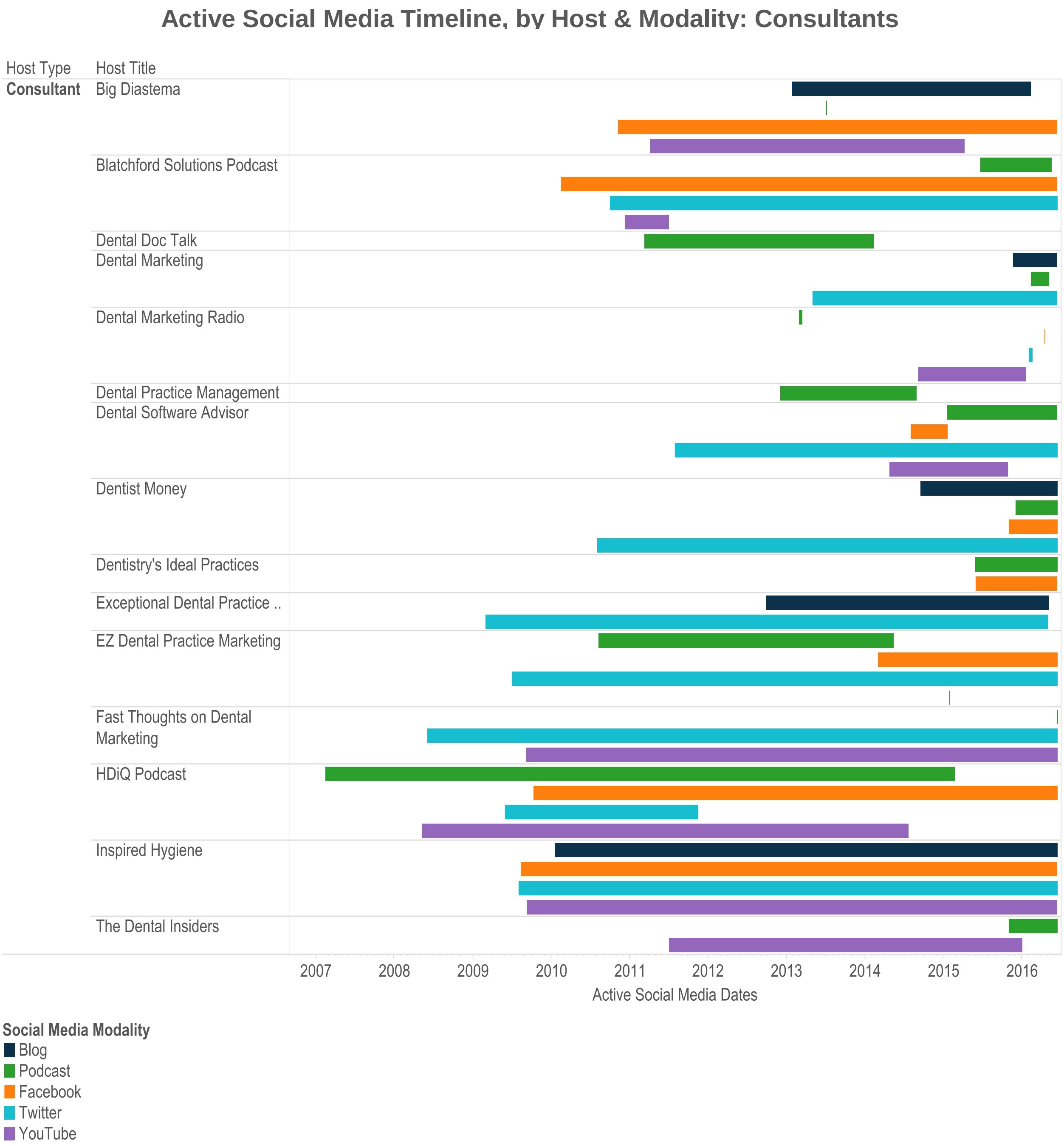
Active Social Media Timeline, by Host & Modality: Consultants
Host Type
Host Title
Consultant
Big Diastema
Blatchford Solutions Podcast
Dental Doc Talk
Dental Marketing
Dental Marketing Radio
Dental Practice Management
Dental Software Advisor
Dentist Money
Dentistry's Ideal Practices
Exceptional Dental Practice ..
EZ Dental Practice Marketing
Fast Thoughts on Dental Marketing
HDiQ Podcast
Inspired Hygiene
The Dental Insiders
2007
2008
2009
2010
2011
2012
2013
2014
2015
2016
Active Social Media Dates
Social Media Modality
Blog
Podcast
Facebook
Twitter
YouTube

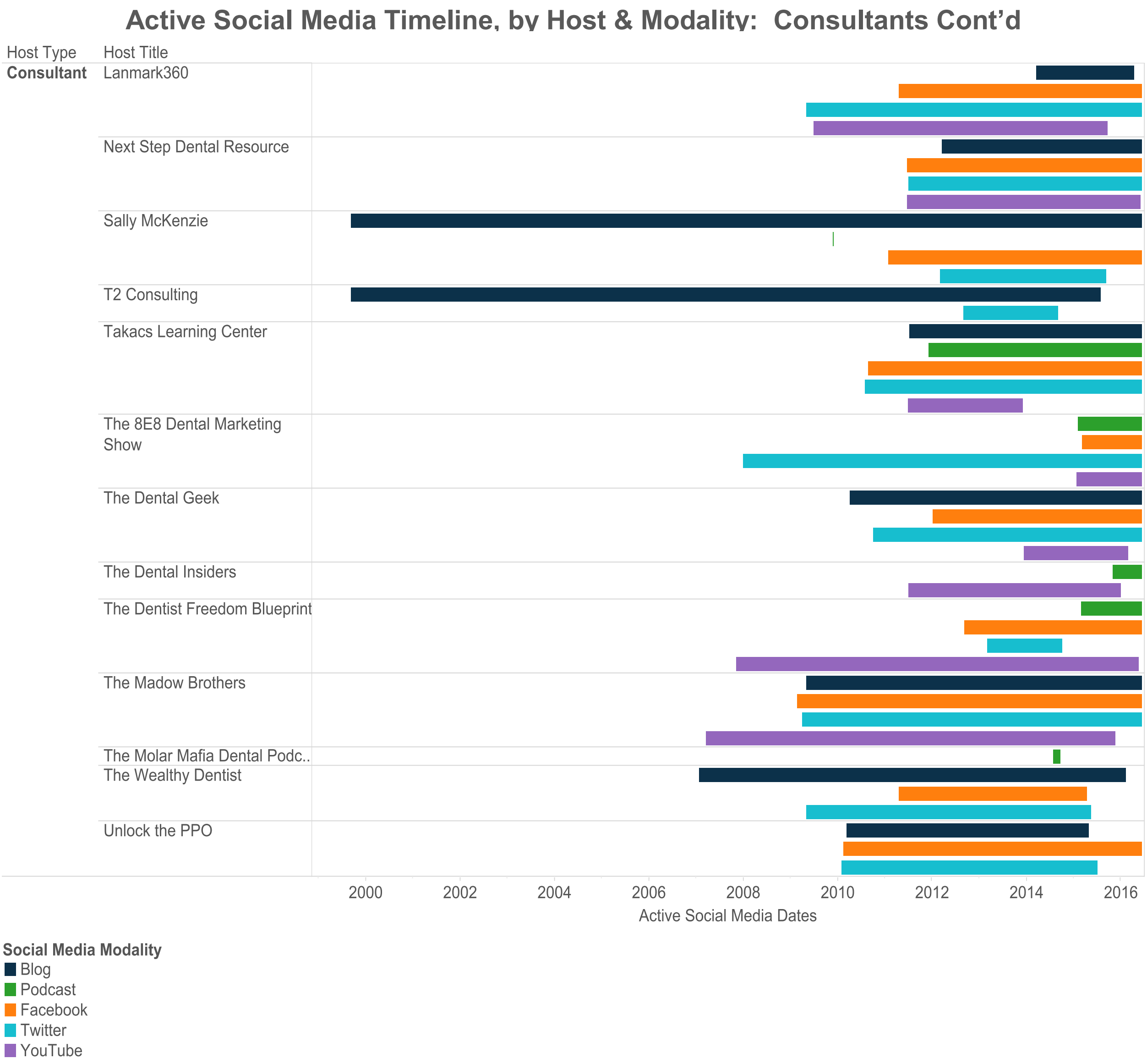
Active Social Media Timeline, by Host & Modality: Consultants Cont'd
Host Type
Host Title
Consultant
Lanmark360
Next Step Dental Resource
Sally McKenzie
T2 Consulting
Takacs Learning Center
The 8E8 Dental Marketing Show
The Dental Geek
The Dental Insiders
The Dentist Freedom Blueprint
The Madow Brothers
The Molar Mafia Dental Podc..
The Wealthy Dentist
Unlock the PPO
2000
2002
2004
2006
2008
2010
2012
2014
2016
Active Social Media Dates
Social Media Modality
Blog
Podcast
Facebook
Twitter
YouTube
