# Supplementary material for: Dental Blogs, Podcasts, and Associated Social Media: Descriptive Mapping and Analysis
Source: J Med Internet Res. 2017 Jul 26;19(7):e269. doi: 10.2196/jmir.7868 (PMC5553003; doi:10.2196/jmir.7868)
Supplement: Multimedia Appendix 3 [file jmir_v19i7e269_app3.pdf]

Topic model of dental blogs (verbatim text)[1]

Legend	Main group, 13 blogs, 4677 posts	High volume, 4 blogs, 9122 posts	DentalEZ, 658 posts	Endo blog, 193 posts	Jablow, 3007 posts	Lee Ann Brady, 478 posts	New Dentist Now, 522 posts	NYC Dentist, 318 posts	Voice of Dental Ed
status/ social	john; guy; kid; talk; thing; stuff; weekend; kind; couple; wife; friday; people; week; day; start	visit; product; announce; information; professional; lead; industry	booth; meeting; exhibit; stop; attend; attendee; show; floor	recall; healing; functional; asymptomatic; fully; apical; month; complete; mta; rct; lesion; tooth; year	product; visit; information; company; industry; service; professional; announce; technology; provide; offer; practice; innovative; market; system	etch; adhesive; dentin; acid; resin; bond; rinse; strength	love; big; hear; fun; week; experience; time; job; lot; school; dr; day; advice	cement; margin; cementation; check; fit; impression; remove; preparation; crown; occlusion; final	share; success; profession; skill; love; learn; involve; career
product announcement	team	I'm; I've; thing	lowspeed; prophy; motor; titan; angle; scaler; buy; highspeed; special	res; clin; periimplant; dent; int; periodontol; maintenance; survival; systematic; periimplantitis; titanium	conclusion; method; abstract; result; study; significant; compare; difference; evaluate; test; aim; group; significantly; journal	time; question; thing; patient; practice; office; year; work; day; team	employment; contract; employee; legal	usual; customary; aetna; conference; reasonable; association; fee; country; insurance; healthcare; pay; base; premium; plan; question	private; residency; center; graduate; time; practice; department; clinic; patient; dr; spend; school; learn; general; year
clinical	composite; restoration; material	drive; storage; backup; gb; hard	screening; cancer; identafi; oral; devicedetection	anatomy; furcation; depth; plan; tract; sinus; palatal; opt; previous; mb; challenge; radiography	cancer	mount; capture; record; model; bite; accurate; impression	conference; annual; meeting	ingredient; fluoride; enamel; dry; child; agent	hygienist; hygiene; job; high; unite; option; college; office; apply; state; good; study
clinical materials or equipment	google; search; website; site	web; site; search	territory; sales; manager	study; publish; article; outcome; al; result; evidence; rate; research	mill; impression; scanner	chroma; shade; match; tab; color; natural	debt; refinance; loan; rate; federal; low	student; center; program; special; school; internship; dr; intern; specialist; skill; opportunity; learn; dorfman; experience	serve; interest; health; service; administration; care; small
oral health	student; school	disease; cancer; periodontal; oral	green; ecofriendly; eda	odontogenic; nasal; sinusitis; rhinosinusitis; ct; maxillary	adhesive; bond; resin; strength; composite; cement	muscle; joint; symptom; occlusion; appliance	south; carolina	article; journal; street; wall; websites; online	clinical; plan; life; assistant
companies/ organizations	patterson; technology	android; app	child; smile; kid; health	barrier; pulpal; regenerative; bleach; internal; development; regeneration	carestream; cs	implant; fixture; bone; tissue; soft; abutment	flow; business; equipment	infection; cavity; jaw	decide; family; care; personal
internet related	oral; health	caesy; patterson	ergonomics; pain; ergonomic	intentional; replantation; mandibular; molar	fda	band; wedge; interproximal; matrix	society; association; asda; leader	bleach; female; satisfy	class; professional; prepare; study; program
computers & imaging	appointment; schedule	carecredit; association; ADA	compressor; osprey; dry; utility	ligament; gingival; periodontal; important	toothpaste; brush; fluoride	provisional; bisacryl; provisionals	thankful	history; periodontal	adea; focus; degree; complete; personal; future; choose; apply
fees, payment	medium; social; facebook	microsoft	win; winner; sweepstake; enter; facebook; chance	crack; fracture; diagnose; vertical	caesy	lip; smile; edge; incisal; face	story; read; full; click; news	abutment; healing	goal; day
management, employees	fee; insurance	image; camera	article; read; issue	fee; provider; medicare; service	dexis; kerr	social; write; content	kid; smile; gkas	dentrix; software; image; management	oral; public; grow
school	skill; face; free	management	webinar; ce; pm	scan; radiation; information; view	mercury; amalgam	cement; retention	ethics; ethical; council; code	orthodontic; brace	sod; virginia
child	canal; root	email; message; send	school	terminology	schein; henry; dentrix	failure	answer; comment; blog	linkow; subperiosteal; jeffrey	process
academic papers	food	kindle; amazon; book	schein; henry	instrument; ultrasonic; separate; file	handpiece	orthodontist; retainer	entity	cast; post; prior	veteran
	video	connection	marketing	repair; perforation; material; defect	lesion; caries	diamond	academy	canker; sore	associate
	child	dexis; sensor	midwinter; chicago	isthmus	university; school; researcher	reduction	veteran	culture; lab	program
	answer; question	social; medium; facebook	everlight; lighting; energy; light	day	sirona; cerec; cadcam	accomplish	licensure; exam	yahoo; google; search; advertiser	include
	email	password; security; protect	glide; air; touch	il; cytokine; maintenance; titanium	association	whiten; bleach	social; medium	surgeon; mm; facial	
	employee	procedure	american; association	bias	child; percent	tubule; sensitivity	medicaid; growth	review	
		cerec	solution	dr	canal; root		ce	water	
		music	wwwdentalezcom; columbia; ramvacâ	fair; cost	antibiotic		award	spend	
		desktop; laptop; pc	hygienist	stem	periodontal; periodontitis; gum; disease		scholarship		
		battery; charge		level	review; evidence				
		program		term	site; web				
					implant; bone				

[1] Spelling and format comes directly from the raw text obtained from the blog sites, in some cases are reduced to the word root for analytical purposes, and therefore may have spelling or other anomalies.
